# Biologics as Therapeutical Agents Under Perspective Clinical Studies for Alzheimer’s Disease

**DOI:** 10.3390/molecules30173479

**Published:** 2025-08-24

**Authors:** Huan Li, Xinai Shen, Beiyu Zhang, Zheying Zhu

**Affiliations:** Division of Molecular Therapeutics and Formulation, School of Pharmacy, The University of Nottingham, Nottingham NG7 2RD, UK

**Keywords:** disease-modifying therapies, RNA therapeutics, immunotherapy, gene therapy, neuroinflammation, neurodegenerative disease

## Abstract

Alzheimer’s disease (AD) is a progressive neurodegenerative disorder characterised by cognitive decline, synaptic loss, and multifaceted pathology involving amyloid-β (Aβ) aggregation, tau hyperphosphorylation, neuroinflammation, and impaired proteostasis. In recent years, biologic therapies, such as monoclonal antibodies, vaccines, antisense oligonucleotides (ASOs), and gene therapies, have gained prominence as promising disease-modifying strategies. In this review, we provide a comprehensive synthesis of current biologic approaches under clinical evaluation for AD. Drawing on data curated from *ClinicalTrials.gov* (as of 2025), we systematically summarise the molecular targets, therapeutic modalities, mechanisms of action, trial phases, and sponsors of over 60 biologic agents. These include Aβ-directed antibodies targeting distinct conformers such as protofibrils, pyroglutamate-modified species, and soluble oligomers; tau-targeted immunotherapies and RNA-based interventions; and emerging platforms focused on neuroimmune modulation, peptide hormones, and microbiota-based strategies. Gene and RNA therapeutics, particularly ASOs and small interfering RNAs (siRNAs) delivered intrathecally or via lipid nanoparticles, are also reviewed for their potential to modulate intracellular targets with high specificity. We also analyse the historical landscape of biologic candidates that failed to reach approval, discussing key reasons for trial discontinuation, including lack of clinical efficacy, safety concerns (e.g., amyloid-related imaging abnormalities), or inadequate biomarker responses. These cases offer crucial insights for refining future drug design. Looking ahead, we highlight major challenges and evolving perspectives in AD biologic therapy: expanding therapeutic targets beyond Aβ and tau, overcoming delivery barriers to the brain, designing prevention-oriented and genetically stratified trials, and navigating regulatory and ethical considerations. Together, these efforts signal a paradigm shift in AD drug development, from symptomatic treatment to mechanism-based precision biologics. By integrating real-time clinical trial data with mechanistic insight, this review aims to inform both translational research and therapeutic innovation in AD.

## 1. Introduction

Dementia refers to a group of neurodegenerative disorders characterised by progressive cognitive decline, memory loss, and impaired daily functioning. Among these, Alzheimer’s disease (AD) is the most common form, accounting for approximately two-thirds of all dementia cases worldwide [1]. According to the World Health Organization, over 50 million people currently live with dementia globally, a number projected to exceed 150 million by 2050. This escalating prevalence not only affects millions of patients and their families but also imposes a significant economic burden, with global healthcare and caregiving costs estimated to reach $1.3 trillion annually [2].

While advancing age remains the most significant risk factor for AD, other contributors include lifestyle factors, cardiovascular comorbidities, and genetic predispositions [3]. At the molecular level, AD is characterised by the accumulation of extracellular β-amyloid (Aβ) plaques and intracellular neurofibrillary tangles (NFTs) composed of p-tau protein [4,5]. These pathological hallmarks disrupt essential cellular processes, leading to oxidative stress [6,7], mitochondrial dysfunction [8], neuroinflammation [9], synaptic loss [10], and ultimately, widespread neuronal degeneration [11], particularly in the hippocampus and cerebral cortex. Such changes begin years before the onset of clinical symptoms and progress silently until substantial cognitive and behavioural impairment becomes evident. Recent advances in fluid and imaging biomarkers, such as plasma phosphorylated tau (pTau217) and digital cognitive assessments, have significantly improved the early prediction of AD, even at preclinical stages [12]. Notably, variants of the Apolipoprotein E (*ApoE*) gene, especially the *ApoE4* allele, have been strongly linked to increased AD risk and earlier onset [13,14,15].

Clinically, AD follows a continuum that is often staged into seven progressive phases, ranging from preclinical, asymptomatic changes to severe dementia and complete loss of independence [16]. Early stages may involve subtle memory lapses or difficulties in word-finding, which eventually evolve into significant impairments in reasoning, recognition, and basic motor functions. While the rate of progression varies between individuals, this framework allows clinicians to evaluate disease severity, monitor progression, and assess the potential efficacy of therapeutic interventions.

Over the past few decades, numerous therapeutic strategies have been explored to modify the course of AD. However, most of the current treatments approved by the Food and Drug Administration (FDA), such as cholinesterase inhibitors [17] and NMDA receptor antagonists [18], provide only temporary symptomatic relief without effectively halting or reversing disease progression. The multifactorial nature of AD pathogenesis, involving protein misfolding, neuroinflammation, impaired clearance mechanisms, and genetic variability, has presented formidable challenges to traditional small-molecule approaches [19].

In light of these limitations, biological therapeutics (biologics) have emerged as promising alternatives for AD treatment. These include mAbs (mAbs), antisense oligonucleotides (ASOs), small interfering RNAs (siRNAs), therapeutic vaccines, and stem cell or gene therapies. Compared to conventional drugs, biologics offer higher target specificity [20] and the potential to intervene directly in pathological mechanisms such as amyloid aggregation, tau hyperphosphorylation, and dysregulated gene expression [21,22]. Moreover, biologics may provide a path toward disease-modifying therapies that can be tailored to an individual’s genetic and molecular profile, an essential feature given the heterogeneous nature of AD.

This review will summarise the current landscape of biologics under clinical investigation for AD, including those that have progressed to human trials, and will also examine past failures to inform future directions. By evaluating both the promise and the challenges of biologics in AD therapy, we aim to provide insight into how these innovative modalities might reshape the future of neurodegenerative disease treatment.

## 2. Pathogenic Mechanisms and the Rationale for Biologic Therapies in AD

### 2.1. AD Pathogenesis: Current Hypotheses

#### 2.1.1. Aβ Plaques and the Amyloid Cascade Hypothesis

The amyloid cascade hypothesis, first proposed in 1992 [23], posits that the accumulation of Aβ peptides in the brain parenchyma is the central initiating event in the pathogenesis of AD [24]. This hypothesis has served as the dominant theoretical framework guiding AD research for over three decades, highlighting Aβ aggregation as a trigger for downstream events such as tau pathology, synaptic dysfunction, neuroinflammation, and neuronal loss.

Aβ peptides are generated from the sequential proteolytic cleavage of amyloid precursor protein (APP) [25], a type I transmembrane glycoprotein that is widely expressed in neuronal tissues. Among the various APP isoforms, APP695, consisting of 695 amino acids, is the predominant form in the brain. APP can be processed via two mutually exclusive pathways: the non-amyloidogenic pathway, which is generally considered non-pathogenic, and the amyloidogenic pathway, which leads to Aβ generation [26].

In the amyloidogenic pathway, β-secretase (BACE1) first cleaves APP at the N-terminus of the Aβ domain, producing a soluble ectodomain fragment sAPPβ and a membrane-retained C-terminal fragment known as C99. The C99 fragment is subsequently cleaved by γ-secretase, a multi-subunit protease complex comprising presenilin-1 or -2 (PSEN1/2), nicastrin, APH-1, and PEN-2. γ-Secretase processes C99 within the transmembrane domain, producing Aβ peptides of variable lengths, most notably Aβ40 and Aβ42 [27]. Aβ40 is the predominant form under normal physiological conditions, whereas Aβ42, though less abundant, is more hydrophobic and aggregation-prone, and is thought to play a key role in plaque formation and neurotoxicity [28].

Initially, fibrillar amyloid plaques, composed largely of aggregated Aβ42, were considered the main neurotoxic agents [29]. However, subsequent studies have highlighted the potential importance of soluble Aβ oligomers, which are capable of impairing synaptic plasticity and neuronal signalling well before plaque deposition. These oligomers may exert toxic effects by disrupting calcium homeostasis, triggering microglial activation, and altering membrane properties [30].

Despite extensive investigation, the precise role of Aβ in AD pathogenesis remains complex and partially unresolved. Nevertheless, the amyloid cascade hypothesis continues to serve as a foundational framework for understanding AD pathology and guiding the development of therapeutic strategies.

#### 2.1.2. Neurofibrillary Tangles and Tau Pathology

NFTs are one of the pathological hallmarks of AD, composed of intracellular aggregates of abnormally phosphorylated tau [31], a microtubule-associated protein critical for maintaining cytoskeletal integrity and facilitating axonal transport. Unlike Aβ, which accumulates extracellularly, tau pathology occurs primarily within neurons and has shown a stronger correlation with cognitive impairment in AD patients [32].

Under physiological conditions, tau is predominantly localized in neuronal axons, where it stabilizes microtubules and supports vesicular and organelle trafficking. Tau is encoded by the *MAPT* gene on chromosome 17, and through alternative mRNA splicing, gives rise to six major isoforms in the adult human brain [33]. These isoforms differ in the number of microtubule-binding repeats (3R or 4R) and N-terminal inserts, contributing to diverse functional and aggregation properties. A balanced expression of 3R and 4R isoforms is essential for normal neuronal function, and disruptions in this ratio are implicated not only in AD but also in other tauopathies, such as Pick’s disease, progressive supranuclear palsy (PSP), and corticobasal degeneration (CBD) [34,35].

In AD, tau undergoes aberrant hyperphosphorylation, predominantly mediated by kinases such as glycogen synthase kinase-3β (GSK-3β), cyclin-dependent kinase 5 (Cdk5), and others [36]. Hyperphosphorylation causes tau to dissociate from microtubules, leading to cytoskeletal destabilization and impaired axonal transport. Detached tau mislocalizes from axons to the somatodendritic compartment, where it accumulates and begins to form paired helical filaments (PHFs) and eventually NFTs [37].

NFT formation is a gradual, multistep process that involves site-specific phosphorylation events. Early phosphorylation occurs at residues such as Thr^231^ and Ser^262^ [38], and as the pathology progresses, additional sites like Thr^181^ [39], Ser^396/404^ [40,41], and Ser^422^ [42] are modified. These phosphorylation patterns have diagnostic relevance, as certain pTau epitopes, especially pTau181 and pTau217, are emerging as sensitive biomarkers detectable in cerebrospinal fluid (CSF) and blood during early disease stages [43,44].

The topographical progression of tau pathology in the brain follows a highly stereotypical pattern, known as Braak staging [45]. In the earliest stages (I–II), NFTs accumulate in the trans entorhinal and entorhinal cortex. During stages III–IV, they spread to the hippocampus and limbic structures, and in the final stages (V–VI), tau pathology invades neocortical areas, corresponding to worsening cognitive deficits. Importantly, the extent and distribution of tau pathology correlate more closely with disease severity and cognitive decline than Aβ plaque burden [46], suggesting that tau may be a key driver of neurodegeneration in AD.

Over the past decade, increasing attention has been given to soluble tau oligomers, which may precede PHF and NFT formation and exert more acute neurotoxic effects [47]. These tau oligomers can disrupt synaptic integrity, induce mitochondrial dysfunction, impair calcium signalling, and trigger proinflammatory responses in glial cells. Unlike mature NFTs, which may be relatively inert by the time of formation, oligomeric tau species are thought to be more dynamic and neurotoxic, similar to soluble Aβ oligomers in the amyloid cascade.

Another critical aspect of tau pathology is its prion-like propagation across neural circuits. Studies have shown that pathological tau can spread across synapses (i.e., trans-synoptic propagation) from one neuron to another, acting as a template that induces misfolding and aggregation of endogenous tau in recipient cells [48]. This mechanism is supported by experimental evidence in both cell-based and animal models and is believed to underlie the anatomical progression observed in Braak staging. Various routes of transmission, including exosome-mediated release, tunnelling nanotubes, and passive leakage from dying neurons, have been proposed [49]. Understanding tau spread dynamics is of growing importance for identifying therapeutic windows and routes of intervention.

Beyond neuronal damage, tau pathology has been shown to interact with other key cellular processes in AD. p-tau can exacerbate oxidative stress, impair autophagic clearance, and contribute to neuroinflammation through activation of microglia and astrocytes [50]. These interactions form a vicious cycle that amplifies neuronal injury and disease progression. Furthermore, tau and Aβ pathologies are not independent. Aβ accumulation can facilitate tau hyperphosphorylation and aggregation [5], although tau pathology can also emerge independently, as seen in primary age-related tauopathy (PART), a condition often found in cognitively normal elderly individuals.

Taken together, the tau hypothesis asserts that abnormal tau phosphorylation, misfolding, and aggregation are central events in AD pathogenesis, particularly in driving neuronal dysfunction and clinical symptoms. While amyloid pathology may initiate the disease, tau appears to mediate the downstream neurodegeneration that directly impairs cognition. This understanding has led to a growing interest in anti-tau therapies, including mAbs, ASOs, and tau aggregation inhibitors, which will be discussed further in later sections of this review. Ultimately, successful AD treatment may require a dual-targeted approach addressing both tau and Aβ pathology to halt or reverse the disease process.

#### 2.1.3. Beyond Amyloid and Tau: Alternative Mechanistic Hypotheses in AD

Despite the central roles of Aβ and tau in AD pathophysiology, a growing body of evidence suggests that these two hallmarks alone cannot fully explain the complex onset and progression of AD [4,5]. Several alternative hypotheses have emerged in recent years, shedding light on other potential therapeutic targets and contributing to a more holistic understanding of the disease. This section discusses five such mechanisms: neuroinflammation, impaired glucose metabolism and insulin signalling, ApoE-related lipid dysregulation, blood–brain barrier (BBB) dysfunction, and mitochondrial dysfunction and oxidative stress.

##### Neuroinflammation and Microglia Activation

The neuroinflammation hypothesis posits that chronic activation of the brain’s innate immune system, particularly microglia and astrocytes, contributes significantly to the pathogenesis of AD [51]. Under physiological conditions, microglia perform immune surveillance, phagocytose debris, and help maintain synaptic homeostasis. However, in AD, microglia are persistently activated by pathological stimuli such as Aβ plaques and hyperphosphorylated tau, leading to sustained release of pro-inflammatory cytokines (e.g., TNF-α, IL-1β, IL-6), chemokines, and reactive oxygen species (ROS). This proinflammatory milieu may exacerbate synaptic dysfunction, neuronal injury, and promote tau pathology propagation.

Genetic evidence strongly supports the neuroinflammation hypothesis. Genome-wide association studies (GWAS) have identified multiple AD-associated variants in immune-related genes, including *TREM2*, *CD33*, and *CR1* [52,53]. For instance, *TREM2* mutations impair microglial phagocytosis and response to injury, leading to inefficient amyloid clearance and increased neurotoxicity. Moreover, positron emission tomography (PET) imaging studies using ligands like [^11^C]-PK11195 or [^18^F]-DPA-714 have demonstrated elevated microglial activation in AD patients, correlating with disease severity and cognitive decline.

Compared to the amyloid cascade hypothesis, neuroinflammation is increasingly recognized as both a downstream amplifier and independent driver of pathology [54]. It may also underlie the failure of anti-amyloid therapies in later disease stages, where inflammation dominates over plaque burden.

From a therapeutic perspective, several anti-inflammatory approaches have been explored. These include small molecules targeting microglial modulators, including AL002 targeting TREM2 [55], or GV-971, a gut-brain axis modulator [56], as well as broad-spectrum immunomodulators like NSAIDs, although clinical efficacy has been limited. The key challenge remains achieving immunomodulation without global immunosuppression and identifying patients in an inflammation-dominant disease window.

In summary, neuroinflammation represents a crucial, genetically validated, and therapeutically actionable pathway in AD. It offers an attractive complement or alternative to Aβ/tau-targeted strategies, particularly in individuals with high innate immune activation signatures.

##### Insulin Resistance and Brain Glucose Hypometabolism Hypothesis

The insulin resistance hypothesis centres on the observation that AD brains exhibit impaired insulin signalling and glucose hypometabolism, features akin to peripheral insulin resistance seen in type 2 diabetes mellitus (T2DM) [57]. This theory suggests that dysfunctional insulin signalling in the central nervous system contributes to synaptic failure, tau hyperphosphorylation, and neurodegeneration, earning AD the moniker “type 3 diabetes” in some literature [58,59].

Glucose hypometabolism is one of the earliest detectable changes in AD, often preceding clinical symptoms by decades [60]. Fluorodeoxyglucose positron emission tomography (FDG-PET) imaging consistently reveals reduced glucose uptake in the posterior cingulate, praecuneus, and parietotemporal cortices of individuals with mild cognitive impairment (MCI) and AD [61]. Mechanistically, insulin regulates key neuronal processes including long-term potentiation, mitochondrial function, and oxidative stress responses. In AD, insulin receptor desensitization and downregulation reduce Akt signalling and enhance GSK3β activity, promoting tau phosphorylation and neuronal death [61,62].

Epidemiological studies show that T2DM increases the risk of developing AD by up to two-fold [63], and insulin resistance correlates with worse cognitive outcomes even in non-diabetic individuals. Postmortem studies of AD brains confirm reduced insulin receptor expression, impaired insulin transport across the BBB, and increased levels of insulin-degrading enzyme (IDE), which competes with Aβ for degradation [59].

Therapeutically, this hypothesis has spurred trials of insulin-sensitizing agents in AD. Intranasal insulin, which bypasses the BBB and delivers insulin directly to the CNS, has shown cognitive benefits in early-phase studies [64], particularly in ApoE4-negative individuals. Other strategies include GLP-1 receptor agonists (e.g., liraglutide, semaglutide) that enhance insulin signalling and exhibit neuroprotective effects in preclinical models. Notably, semaglutide is currently being evaluated in large phase 3 trials for AD [65].

However, the complexity of CNS insulin signalling and its interplay with amyloid and tau pathways poses challenges [1]. While the hypothesis is supported by robust metabolic, clinical, and imaging data, therapeutic translation remains in its infancy. Stratification by metabolic phenotype and genotype (e.g., *ApoE4* status) may be key to unlocking its clinical utility.

##### Lipid Metabolism and ApoE Pathway Dysfunction Hypothesis

Disruptions in lipid metabolism, particularly involving ApoE, constitute a major non-amyloid, non-tau hypothesis in AD pathogenesis [14]. ApoE is the principal lipid transporter in the brain, responsible for redistributing cholesterol and phospholipids among neurons and glia [66]. Among its three major isoforms, including *ApoE2*, *ApoE3* and *ApoE4*, *ApoE4* is the strongest genetic risk factor for late-onset AD (LOAD), increasing disease risk by up to 12-fold in homozygotes compared to the neutral *ApoE3* allele [13,67,68]. *ApoE2*, conversely, appears protective [69].

The ApoE4 isoform impairs lipid homeostasis, synaptic repair, and Aβ clearance [15,70]. Structurally, ApoE4 exhibits a domain interaction that alters its lipid-binding properties, leading to reduced lipidation by ATP-binding cassette transporter A1 (ABCA1) [15]. This results in less stable ApoE-containing lipoproteins and impaired transport of cholesterol to neurons, which depend on lipid supply for membrane remodelling and synaptic plasticity. ApoE4 also facilitates aggregation and impaired clearance of Aβ through reduced affinity for lipoprotein receptors such as LRP1, thereby contributing to amyloid accumulation [71].

Importantly, ApoE also influences tau pathology and neuroinflammation [72]. In mouse models, ApoE4 exacerbates tau-induced neurodegeneration independent of Aβ, and promotes a more neurotoxic microglial phenotype. Additionally, ApoE4 alters mitochondrial metabolism, increases oxidative stress, and disrupts lipid raft composition [73], further impairing receptor signalling at synapses.

Therapeutically, targeting ApoE and associated lipid pathways offers a multifaceted approach. Strategies under investigation include enhancing ApoE lipidation via liver X receptor (LXR) or retinoid X receptor (RXR) agonists (e.g., bexarotene) [74], gene editing to convert *ApoE4* to *ApoE3* [75], and ASOs [76] or siRNAs [77] designed to suppress ApoE4 expression. Small molecules that disrupt ApoE4 domain interaction (e.g., PH002) are also in preclinical development [78]. However, targeting ApoE poses significant challenges due to its dual roles in peripheral and central lipid metabolism, and the need to preserve ApoE2/3 function while mitigating ApoE4-specific toxicity.

In summary, the lipid metabolism hypothesis, anchored by ApoE biology, integrates genetic, biochemical, and neuropathological evidence, offering one of the most compelling non-amyloid pathways in AD. It is particularly promising for preventive strategies in ApoE4 carriers, who represent over 50% of LOAD cases.

##### BBB Dysfunction Hypothesis

The BBB dysfunction hypothesis posits that structural and functional breakdown of the BBB contributes to the initiation and progression of AD [79]. The BBB, composed of tightly connected endothelial cells, pericytes, astrocytic endfeet, and extracellular matrix, is essential for maintaining central nervous system (CNS) homeostasis by regulating molecular transport, restricting immune cell entry, and clearing neurotoxic waste, including Aβ.

In aging and AD, increasing evidence points to early and progressive BBB disruption [80]. Dynamic contrast-enhanced MRI (DCE-MRI) and PET imaging studies have revealed elevated BBB permeability in cognitively normal individuals at risk for AD, especially ApoE4 carriers, as well as in patients with MCI and AD. Notably, BBB breakdown in the hippocampus correlates with cognitive decline independently of amyloid or tau burden, suggesting a distinct pathogenic role [81].

Mechanistically, BBB dysfunction impairs Aβ clearance via transporters such as LRP1 and P-glycoprotein (P-gp), which mediate efflux of Aβ from brain to blood [82]. Simultaneously, upregulation of receptor for advanced glycation end-products (RAGE) facilitates influx of peripheral Aβ and inflammatory mediators [83]. Pericyte loss and endothelial degeneration further compromise barrier integrity, enabling entry of plasma proteins (e.g., fibrinogen), immune cells, and oxidative stressors into the CNS, leading to neuroinflammation, synaptic toxicity, and tau hyperphosphorylation.

Genetic and transcriptomic studies have begun to link AD-associated risk loci (e.g., ApoE4, CLDN5, PICALM) to BBB-related pathways. ApoE4, in particular, disrupts cerebrovascular function by activating the CypA–MMP9 pathway in pericytes [84], resulting in tight junction degradation and increased barrier leakage.

Therapeutic efforts targeting BBB integrity are still in early stages but gaining momentum [85]. Strategies under exploration include:

- Enhancing efflux transporter function (e.g., LRP1 upregulation),
- Inhibiting RAGE-mediated Aβ influx,
- Modulating endothelial inflammation (e.g., via anti-VCAM1 agents),
- Promoting pericyte survival and vascular stability.

BBB dysfunction also has major implications for drug delivery in AD. Understanding BBB permeability dynamics may guide the development of CNS-targeted delivery systems, including lipid nanoparticles, focused ultrasound, and intranasal formulations.

In conclusion, BBB breakdown is not merely a late consequence of neurodegeneration but may be an initiating event, especially in genetically vulnerable individuals. Recognizing cerebrovascular dysfunction as a therapeutic target opens new avenues for early intervention in AD.

##### Mitochondrial Dysfunction and Oxidative Stress Hypothesis

Mitochondrial dysfunction and oxidative stress have long been implicated in the pathogenesis of AD. This hypothesis proposes that impaired mitochondrial bioenergetics and increased production of ROS contribute to synaptic degeneration, neuronal death, and the progression of AD, potentially as both upstream and downstream events relative to Aβ and tau pathology [86,87].

Mitochondria are essential for ATP production, calcium buffering, redox regulation, and apoptotic control in neurons. In AD, multiple mitochondrial defects have been observed, including [88]:

- Reduced activity of key oxidative phosphorylation (OXPHOS) enzymes (e.g., cytochrome c oxidase, complex I),
- Mitochondrial DNA (mtDNA) mutations and deletions,
- Abnormal mitochondrial morphology and dynamics (fission/fusion imbalance),
- Disrupted transport along axons and dendrites.

These defects are often accompanied by elevated ROS levels, leading to oxidative damage of lipids, proteins, and nucleic acids. Postmortem analyses of AD brains reveal extensively oxidative modifications (e.g., 4-HNE, 8-OHdG) even in early disease stages [89]. Notably, Aβ and tau can both localize to mitochondria and further impair function, Aβ by binding to mitochondrial membranes and disrupting respiratory chain activity [90], and p-tau by interfering with mitochondrial transport and axonal energy supply [91].

Moreover, ApoE4 has been linked to mitochondrial fragmentation and bioenergetic deficits. In vitro and in vivo studies suggest that ApoE4-expressing neurons have lower mitochondrial membrane potential, decreased ATP output, and increased vulnerability to oxidative insults, potentially compounding other pathogenic pathways [92].

Therapeutic strategies targeting mitochondrial dysfunction have focused on [93]:

- Antioxidants (e.g., vitamin E, coenzyme Q10, MitoQ),
- Mitochondria-targeted peptides (e.g., SS-31/Elamipretide),
- NAD^+^ precursors (e.g., nicotinamide riboside) to support mitochondrial biogenesis,
- Agents that enhance mitophagy and mitochondrial dynamics (e.g., urolithin A).

Although antioxidant therapies have yielded disappointing results in large clinical trials [94], these failures may reflect poor CNS penetration, lack of patient stratification, or intervention at too late a stage. Newer approaches targeting mitochondrial resilience at early or even preclinical AD stages are under active investigation.

In summary, mitochondrial dysfunction represents a convergence point for genetic, metabolic, and protopathic insults in AD. While not traditionally considered a primary driver, it may be a crucial amplifier of neurodegeneration and thus a viable therapeutic target.

##### Gut-Brain Axis Hypothesis

Beyond amyloid, tau, vascular, and metabolic hypotheses, the gut–brain axis has emerged as an important contributor to AD pathogenesis. The gut–brain axis describes the bidirectional communication between the gastrointestinal microbiota and the central nervous system through neural, immune, endocrine, and metabolic pathways [95]. Growing evidence indicates that dysbiosis of gut microbiota may exacerbate neuroinflammation, amyloid deposition, and cognitive decline in AD [96,97]. Several studies have reported altered gut microbial composition in AD patients, including reduced diversity and changes in the relative abundance of phyla such as Firmicutes and Bacteroidetes [98,99,100]. Notably, AD-associated microbiota shifts are often accompanied by decreased production of short-chain fatty acids (SCFAs), such as butyrate, which normally exert anti-inflammatory and neuroprotective effects [101].

Mechanistically, microbial metabolites play a critical role. SCFAs and tryptophan metabolites influence microglial maturation and immune responses, while secondary bile acids have been implicated in promoting neurotoxicity and oxidative stress [102]. Dysbiosis also compromises gut barrier integrity, leading to systemic endotoxemia and chronic peripheral inflammation, which in turn aggravates BBB dysfunction and neuroinflammatory cascades [103]. Animal models provide compelling evidence that transplantation of microbiota from AD patients into germ-free mice can accelerate amyloid accumulation and cognitive deficits, while probiotic supplementation has been shown to improve memory performance and synaptic plasticity in transgenic AD models [104,105].

Importantly, the gut–brain axis interacts with established AD pathways. Dysbiotic microbiota can modulate Aβ clearance by influencing systemic immune tone [106], promote tau hyperphosphorylation via pro-inflammatory cytokines [97], and alter ApoE lipid metabolism through bile acid signaling [107]. Thus, the gut–brain axis hypothesis provides a unifying framework that integrates immune, metabolic, and neurovascular dysfunction. It also highlights the possibility that gut-targeted interventions could serve not only as adjunctive therapies but also as preventive measures in at-risk populations.

### 2.2. Genetic Risk Factors in AD

AD is broadly classified into two major types based on age of onset and genetic etiology: early-onset familial AD (EOAD), which typically manifests before the age of 65 and accounts for less than 1% of cases, and LOAD, which constitutes the vast majority [108]. EOAD is usually inherited in an autosomal dominant fashion and caused by rare, high-penetrance mutations [109]. In contrast, LOAD is influenced by a complex interplay of common genetic risk variants, environmental factors, and aging [110]. Over the past two decades, extensive genetic studies have uncovered both causative mutations and susceptibility loci that contribute to AD risk, shaping our current understanding of disease mechanisms and therapeutic targeting.

#### 2.2.1. APP

Mutations in the *APP* gene were the first to be identified as causative of familial AD [111]. *APP* encodes a type I transmembrane protein that undergoes sequential cleavage by β-secretase and γ-secretase to produce Aβ peptides. Pathogenic mutations, especially those near the β- or γ-secretase cleavage sites, either increase total Aβ production or shift the balance toward the more aggregation-prone Aβ42 isoform [112]. For example, the Swedish mutation (KM670/671NL) enhances β-secretase cleavage [113], while the London mutation (V717I) affects γ-secretase processing [114]. These alterations accelerate plaque formation and neurotoxicity, often leading to disease onset in the 40 s or 50 s. Although *APP* mutations are rare, their discovery was instrumental in forming the amyloid cascade hypothesis and in the development of many amyloid-targeting therapies.

#### 2.2.2. PSEN1/PSEN2

*Presenilin 1* (*PSEN1*) and *Presenilin 2* (*PSEN2*) encode catalytic subunits of the γ-secretase complex that cleaves APP during Aβ generation [115]. Mutations in *PSEN1* are the most common cause of EOAD, accounting for over 70% of familial cases [116]. These mutations often result in altered γ-secretase activity, producing longer, more toxic Aβ42 peptides. In addition to affecting amyloid processing, *PSEN1* mutations may disrupt calcium homeostasis, lysosomal function, and neuronal development [117]. *PSEN2* mutations are less frequent but similarly impair γ-secretase function and can lead to early- or mid-life onset dementia [118]. Unlike *APP* mutations, which affect substrate availability, presenilin mutations primarily affect enzymatic cleavage, emphasizing the diversity of pathological mechanisms even within the same molecular pathway.

#### 2.2.3. ApoE

The *ApoE* gene is the strongest and most prevalent genetic risk factor for LOAD [13,119]. *ApoE* exists in three isoforms, encoded by corresponding alleles. Possession of one copy of the *E4* allele increases AD risk 2–3 fold, while two copies can raise it by over 10-fold. In contrast, *ApoE2* appears to confer protection. *ApoE* influences several aspects of brain function, including lipid transport, synaptic maintenance, and immune response. Critically, *ApoE4* impairs Aβ clearance through reduced interaction with lipoprotein receptors such as LRP1, promotes neuroinflammation, enhances tau pathology, and compromises BBB integrity. Its broad impact on multiple AD-relevant pathways has made *ApoE4* a prominent target in both genetic and pharmacologic research, including ongoing efforts to silence or modulate its expression via RNA-based therapeutics [120,121]. These genetic findings further reinforce the lipid metabolism and ApoE pathway dysfunction hypothesis discussed in Section 2.1, highlighting *ApoE4* as both a genetic and mechanistic driver of AD pathology.

#### 2.2.4. TREM2

Among the AD risk genes identified beyond the classical *APP*, *PSEN1/2*, and *ApoE*, triggering receptor expressed on myeloid cells (*TREM2*) has a relatively large impact on AD risk compared to other common variants, with odds ratios ranging from 2 to 4 in carriers of the R47H mutation [122,123]. TREM2 is a transmembrane receptor expressed primarily on microglia in the CNS and is involved in sensing tissue damage, modulating immune responses, and regulating phagocytic activity. Rare heterozygous missense mutations, most notably the R47H variant, increase the risk of LOAD by approximately 2- to 4-fold [124]. Functional studies have shown that impaired *TREM2* signalling leads to defective microglial clustering around amyloid plaques, diminished clearance of cellular debris and toxic proteins, and a shift toward a pro-inflammatory microglial phenotype [125]. This altered immune environment may exacerbate neuronal injury and facilitate the propagation of tau pathology. As a result, *TREM2* is now being actively pursued as a therapeutic target, particularly for biologic agents that aim to enhance microglial protective function or restore phagocytic capacity.

Beyond *TREM2*, large-scale GWAS have uncovered a growing list of common genetic variants associated with increased AD risk. These include *clusterin* (*CLU*), *bridging integrator 1* (*BIN1*), *ABCA7*, *CD33*, *PICALM*, and *SORL1* [126], among others. Although each of these loci individually confers only a modest increase in disease risk, collectively they implicate several key biological pathways. For example, *CLU* and *ABCA7* are involved in lipid metabolism and may influence Aβ transport and clearance [127,128]; *BIN1* and *PICALM* regulate endocytosis and intracellular trafficking, including tau vesicle sorting [129,130]; *CD33*, like *TREM2*, modulates innate immune responses through microglial signalling [131]; and *SORL1* controls *APP* sorting in the endosomal-lysosomal system, thereby affecting Aβ production [132]. These findings suggest that AD pathogenesis arises not from a single dysfunctional pathway, but rather from a convergence of disrupted immune regulation, lipid homeostasis, and vesicular trafficking, all of which interact with and potentially exacerbate amyloid and tau pathology. This systems-level view is critical for identifying combination therapies and understanding the variable clinical presentations of the disease.

The expanding catalogue of AD-associated genes has important implications for therapeutic innovation, particularly in the realm of biologic agents. High-expression or loss-of-function variants in genes such as *ApoE*, *TREM2*, and *SORL1* provide clear molecular targets for biologics like ASOs, siRNAs, and mAbs. For instance, agonistic antibodies activating TREM2 signalling aim to boost microglial protective functions in carriers of impaired alleles [133].

Moreover, understanding patient-specific genotypes allows for personalized treatment selection and trial stratification, paving the way for precision neurology. As biologic therapeutics become increasingly modular and gene-specific, integrating genetic risk profiles into drug development pipelines will likely enhance both efficacy and safety. Thus, the genetic underpinnings of AD are not only foundational for understanding disease mechanisms but also serve as critical entry points for next-generation biologic therapies.

### 2.3. Biologics as Therapeutics Agents

#### 2.3.1. Overview and Classification of Biologic Therapeutics

Biologic therapeutics, commonly referred to as biologics, are a diverse class of medicinal products derived from living organisms or produced through recombinant technologies. Unlike small-molecule drugs, which are chemically synthesized and generally low in molecular weight, biologics include proteins, nucleic acids, viral vectors, and living cells, offering the ability to target disease mechanisms with high specificity and biological relevance. As our understanding of AD has evolved from a proteinopathy-centric model to one involving multifaceted, interconnected pathological processes, biologics have emerged as promising therapeutic modalities with the potential to address the molecular complexity of AD more precisely than conventional drugs [21].

Biologics can be broadly categorized based on their molecular composition and therapeutic mechanism. The most widely studied class in AD is mAbs, which are designed to bind specifically to Aβ or tau proteins to neutralize, promote clearance, or prevent their aggregation. FDA-approved agents such as Aducanumab and Lecanemab fall into this category [134], as do tau-targeting antibodies like Semorinemab and Gosuranemab in clinical development. A second major category includes nucleic acid-based therapeutics, such as ASOs and siRNAs. These agents modulate gene expression at the RNA level, offering a powerful approach to silence toxic gene products such as APP, tau (MAPT), or ApoE4 without altering the underlying DNA sequence.

Another emerging class of biologics is therapeutic vaccines, designed to stimulate the host immune system to recognize and clear pathological proteins [135]. Examples include active Aβ vaccines like UB-311, as well as tau-targeted immunization strategies in early-stage trials. In addition, cell-based therapies, including mesenchymal stem cells (MSCs) and neural stem cells (NSCs), have been explored for their ability to modulate inflammation, secrete neurotrophic factors, and possibly replace lost neurons [136,137]. Finally, gene therapy vectors such as adeno-associated viruses (AAVs) are being investigated as delivery vehicles for neuroprotective genes, such as nerve growth factor (NGF) or brain-derived neurotrophic factor (BDNF), to promote long-term neuronal support and plasticity [138].

Together, these diverse biologic modalities offer a rich toolkit for targeting different facets of AD pathology, from protein accumulation and immune dysfunction to synaptic failure and neurodegeneration. As technological advances improve the design, stability, and delivery of biologics, their therapeutic potential continues to expand, opening new avenues for disease modification and personalized treatment strategies.

#### 2.3.2. Advantages over Traditional Small-Molecule Drugs

The clinical approvals of Aducanumab, Lecanemab, and Donanemab and all biologics as disease modified therapeutics provide real-world evidence supporting the principle that biologics can achieve amyloid clearance and modest slowing of cognitive decline, effects not achieved with small molecules [139,140]. While traditional small-molecule drugs have been the mainstay of pharmacological treatment for neurodegenerative diseases, including AD, their limitations have become increasingly evident in the context of AD’s complex pathology. Small molecules are typically designed to bind to active sites on enzymes or receptors and often lack the specificity and modularity required to address multi-layered pathological processes such as protein aggregation, neuroinflammation, and gene dysregulation. In contrast, biologic therapeutics offer several distinct advantages that make them particularly well-suited for addressing the challenges posed by AD [21].

First and foremost, biologics demonstrate high molecular specificity, allowing them to selectively bind to pathological forms of proteins, such as misfolded Aβ oligomers or hyperphosphorylated tau, while sparing normal physiological forms. mAbs, for instance, can be engineered to target conformational epitopes or post-translationally modified proteins, a level of precision that is difficult to achieve with small molecules [141]. This specificity reduces off-target toxicity and enhances therapeutic selectivity, an especially critical feature when treating diseases of the CNS where widespread cellular targets could cause unintended side effects.

Second, biologics can target disease mechanisms that are inaccessible to small molecules, particularly in the realm of gene regulation and protein-protein interactions. Nucleic acid-based therapeutics such as siRNAs and ASOs enable the post-transcriptional silencing or modulation of genes implicated in AD, including *ApoE4*, *APP*, *BACE1*, or tau [76,142,143,144]. These approaches go beyond symptom management and offer the possibility of modifying disease progression by directly reducing the expression of pathogenic drivers at the RNA level.

Biologics also offer longer duration of action and often require less frequent dosing [21]. For example, antibody therapies typically have half-lives ranging from several days to weeks, which facilitates monthly or even less frequent administration, an advantage for chronic diseases like AD that require long-term intervention. Moreover, the development of sustained-delivery systems (e.g., AAV vectors, lipid nanoparticles) further extends the pharmacological window of biologic therapies and enhances patient adherence [145].

Another advantage lies in the advancement of delivery technologies, including intranasal [146,147], intrathecal, and nanoparticle-assisted delivery systems that improve bioavailability to the CNS. These approaches help to overcome the BBB, a major obstacle for both small molecules and biologics, but one where biologics now show increasing promise due to innovations in carrier engineering, such as cell-penetrating peptides and receptor-mediated transcytosis systems.

Finally, the design of biologics is often highly modular, allowing rapid adaptation based on emerging genetic or biomarker data. This is particularly valuable in AD, where patient subtypes defined by *ApoE* genotype, inflammation profiles, or Aβ/tau burden may benefit from tailored interventions. Such adaptability aligns with the broader movement toward precision medicine in neurodegenerative diseases.

In summary, while small molecules remain useful for symptomatic relief and certain well-defined targets, biologics provide a superior therapeutic platform for disease modification, mechanistic precision, and personalized application in AD.

#### 2.3.3. Mechanistic Compatibility with AD Pathology

One of the most compelling reasons to pursue biologic therapeutics in AD lies in their mechanistic compatibility with the underlying pathophysiology. AD is not a single-pathway disease, but rather a multifactorial disorder involving protein misfolding, chronic inflammation, synaptic loss, metabolic disruption, and gene-environment interactions. Biologics, due to their structural diversity and programmable specificity, are uniquely suited to engage with this biological complexity [86].

At the core of AD pathology is the accumulation of Aβ plaques and NFTs composed of hyperphosphorylated tau. mAbs targeting Aβ, such as Lecanemab and Donanemab, are designed to recognize aggregated or protofibrillar forms of Aβ, facilitating their clearance via Fc receptor-mediated microglial phagocytosis. Similarly, anti-tau antibodies like Semorinemab and BIIB076 aim to neutralize extracellular tau species that propagate pathology between neurons [148,149]. These biologics directly engage with the central proteinopathy of AD and reflect a strategy not easily achievable with small molecules, which often struggle to distinguish pathological versus physiological protein forms.

Beyond targeting extracellular aggregates, biologics also address intracellular and genetic contributors to AD [150]. For example, ASOs and siRNAs allow precise knockdown of overexpressed or mutated genes, such as *MAPT* (tau) [151], *APP* [152], or *ApoE4* [76], thereby reducing the production of pathogenic proteins upstream of plaque or tangle formation. These gene-silencing approaches are particularly appealing for treating familial AD cases involving known mutations or for modulating expression of risk alleles in sporadic AD, such as in ApoE4 carriers.

Biologics also show compatibility with neuroimmune and neurovascular dimensions of AD pathology. Antibodies targeting microglial receptors like TREM2 aim to restore beneficial microglial responses, enhancing amyloid clearance and promoting a neuroprotective immune profile [133]. Similarly, modulating receptors such as CD33 or enhancing BBB stability via endothelial-targeted agents offers a way to intervene in the inflammatory and vascular cascades that often precede or exacerbate cognitive decline [153].

Furthermore, the use of gene therapy vectors, such as AAVs, to deliver neuroprotective factors like NGF or BDNF aligns well with the neurotrophic deficits observed in AD [138]. These factors support synaptic plasticity, neuronal survival, and mitochondrial function, addressing downstream consequences of chronic pathology. This regenerative approach goes beyond disease suppression and toward restoring lost function, an essential goal in advanced-stage AD.

The modularity of biologics also allows integration with biomarker-guided treatment strategies. For instance, PET or CSF biomarkers indicating high tau burden, neuroinflammation, or ApoE4 status can guide the selection of appropriate biologic agents, ushering in a new era of precision neurology. As our understanding of AD endophenotypes evolves, biologics can be engineered or combined to target specific pathological signatures, potentially enhancing both efficacy and safety.

In conclusion, biologic therapeutics not only offer structural and pharmacokinetic advantages over small molecules but also align closely with the multi-axis nature of AD pathology. Their ability to precisely intervene in genetic, protein, and immune pathways makes them ideal candidates for disease-modifying strategies in AD.

## 3. Biologic Therapeutics in Clinical Trials for AD

Over the past two decades, the clinical development landscape for AD has undergone a dramatic transformation, shifting from predominantly symptomatic treatments toward mechanism-based disease-modifying therapies (DMTs) [154]. This evolution has been driven by increasing recognition of the multifactorial nature of AD and the failure of traditional small-molecule drugs to halt or reverse disease progression. In this context, biologic therapeutics have emerged as a central focus of contemporary AD clinical trials, offering diverse modalities, from mAbs to RNA-based drugs, gene therapies, and immunomodulatory strategies, that target specific aspects of disease pathology with unprecedented precision, as summarised in Table 1.

The most clinically advanced class of biologics in AD are mAbs targeting Aβ, with Aducanumab, Lecanemab, and Donanemab each reaching Phase III trials or receiving conditional approval [155,156,157]. These agents represent a paradigm shift in AD treatment strategy, attempting to modify the disease course by directly engaging with protein aggregation, the hallmark pathology of AD. Despite mixed results and controversies, particularly surrounding efficacy endpoints and side effects such as amyloid-related imaging abnormalities (ARIA) [134,158,159], these trials have paved the way for regulatory pathways and biomarker-based patient selection strategies.

Beyond mAbs, RNA-based biologics such as ASOs and siRNAs are being tested in early-phase trials to downregulate genes like *MAPT* (tau) [160], *APP* [143,152], or *ApoE4* [76], representing a shift from extracellular to intracellular and upstream intervention. At the same time, therapeutic vaccines (e.g., UB-311) [161] and gene therapies using AAV vectors to deliver *ApoE2* mRNA [121] or immune modulators are being explored for their long-term disease-modifying potential.

However, the high failure rate of AD trials remains a sobering reality. A recent review of the AD drug development pipeline revealed that over 90% of biologic candidates fail in Phase II or III trials [162], often due to insufficient efficacy, poor biomarker stratification, or late intervention in the disease course. These challenges have spurred a new generation of trial designs incorporating biomarker-based inclusion criteria (e.g., PET-confirmed Aβ or tau burden), earlier disease stages (MCI or even preclinical AD), and genotype-stratified cohorts to improve trial sensitivity and relevance.

As of now, biologics represent the most active and promising segment of the AD therapeutic pipeline, with dozens of trials ongoing worldwide. The following sections will dissect the major categories of biologic therapeutics under clinical investigation and analyse their progress, challenges, and future prospects.

### 3.1. Passive Immunotherapy: mAbs Against Aβ and Tau

mAbs targeting Aβ or tau represent the most clinically advanced class of biologics for AD. These passive immunotherapies have yielded both landmark approvals and high-profile failures, reflecting the critical influence of antigen selectivity, target aggregation state, and disease stage at administration.

#### 3.1.1. Anti-Aβ mAbs

Early-generation antibodies such as Solanezumab and Bapineuzumab were designed based on distinct pathogenic assumptions about Aβ toxicity. Solanezumab binds to monomeric Aβ in the brain interstitial fluid, aiming to sequester soluble peptides and prevent oligomer formation [163]. However, multiple large trials (e.g., EXPEDITION, DIAN-TU) showed no meaningful reduction in amyloid burden or cognitive benefit. In contrast, Bapineuzumab and Gantenerumab targeted fibrillar Aβ within plaques and achieved some plaque clearance but failed to show clinical efficacy and induced high rates of ARIA, especially cerebral edema (ARIA-E) and microhemorrhages (ARIA-H) [164,165].

The recent approvals of Lecanemab and Donanemab mark a shift toward antibodies targeting more toxic Aβ intermediates, such as protofibrils and pyroglutamate-modified Aβ species [156]. Both drugs demonstrated significant reductions in amyloid PET signal and modest slowing of cognitive decline (~27–35%) in early-stage AD populations (CDR 0.5) [166]. Notably, their trials (CLARITY-AD, TRAILBLAZER-ALZ 2) incorporated rigorous biomarker screening and early disease-stage enrolment, addressing past failures where treatment began too late.

Despite these advances, key limitations remain. ARIA incidence remains substantial, particularly in ApoE4 homozygotes [167], and intravenous dosing with frequent MRI monitoring presents logistical and cost-related challenges. Moreover, the clinical benefits are statistically significant but arguably clinically modest, raising questions about long-term real-world value [168].

Mechanistically, these results reaffirm Aβ as a therapeutically tractable but temporally limited target, most effective in preclinical or prodromal stages, and unlikely to reverse downstream pathology once tau and neurodegeneration are established. As such, anti-Aβ antibodies may serve best as early-intervention tools, perhaps in combination with tau- or inflammation-targeted therapies.

#### 3.1.2. Anti-Tau mAbs

Tau-targeted immunotherapy has emerged as a key strategy in AD, driven by the observation that tau pathology correlates more closely with cognitive impairment than Aβ deposition [169]. Therapeutic approaches have focused on either passive immunization using mAbs or active immunization through vaccines, aiming to neutralize pathogenic tau species and halt their propagation.

mAbs against tau, such as Semorinemab, Gosuranemab, Zagotenemab, and Tilavonemab, were designed to bind extracellular forms of tau and thereby prevent its “prion-like” spread between neurons [170]. While this concept is supported by experimental models, clinical trials have yielded disappointing results. In a Phase 2 trial, Semorinemab failed to produce cognitive benefits in moderate AD, despite lowering plasma tau levels [171]. Similarly, Gosuranemab, Zagotenemab and Tilaconemab showed no significant effect on either biomarkers or cognition in placebo-controlled studies [172,173].

Several factors may explain these failures. First, the extracellular tau pool represents only a fraction of total pathogenic tau, and antibodies have limited access to the cytosol, where toxic aggregates accumulate. Second, tau pathology is often already widespread at the time of clinical diagnosis, limiting the potential for antibodies to block propagation. Finally, tau exists in multiple conformational and phosphorylation states, and current antibodies may lack sufficient selectivity or affinity for the most toxic forms.

### 3.2. Active Immunotherapy: Vaccines Targeting Aβ and Tau

Active immunotherapy, or therapeutic vaccination, represents a distinct subclass of biologics that seeks to stimulate the body’s own immune system to recognize and neutralize pathological proteins involved in AD, primarily Aβ and tau. Unlike mAbs that require repeated administration, vaccines may offer longer-lasting effects, lower cost, and improved accessibility, making them particularly attractive for population-wide or preventive strategies.

The earliest efforts at Aβ vaccination, such as AN1792, were halted due to meningoencephalitis in a subset of participants, attributed to off-target T cell activation [174]. This outcome underscored the need for safer epitope design and T cell-independent formulations. More recent vaccine candidates, most notably UB-311, have addressed these concerns through rational antigen engineering [161].

UB-311, a synthetic peptide vaccine designed to elicit polyclonal antibodies against oligomeric Aβ, utilizes helper T-cell epitopes to boost B-cell responses while minimizing pro-inflammatory risk [161]. In a Phase 2a trial, UB-311 was well-tolerated and generated sustained Aβ-specific antibody titers. [175] Although the study was not powered for clinical efficacy, it showed promising trends toward cognitive stabilization and reduced neuropsychiatric symptoms. UB-311 has since advanced to Phase 2b, representing one of the most clinically mature Aβ vaccine candidates to date.

On the tau side, several active immunization strategies are in development. ACI-35 is a liposome-based vaccine targeting p-tau epitopes, designed to induce conformationally selective antibody responses [170,176]. Phase 1b/2a data show robust immunogenicity, with no major safety signals [177]. Another candidate, AADvac1, elicits antibodies against truncated, misfolded tau forms implicated in early tangle formation. Though AADvac1 completed a Phase 2 trial without meeting primary endpoints [178,179], exploratory analyses suggested a potential cognitive benefit in biomarker-positive subgroups.

Despite these advances, challenges remain. One major concern is the limited ability of antibodies, endogenous or exogenous, to penetrate neurons and access intracellular tau aggregates, which may limit efficacy in symptomatic AD. Additionally, heterogeneity in patient immune responses, the need for booster dosing, and delayed onset of action present practical hurdles. Immunotherapies against tau have thus far not replicated the relative success of anti-Aβ antibodies, and their failure to improve clinical outcomes suggests the need to reconsider both the target (e.g., intracellular vs. extracellular tau) and the delivery mechanism. While immunotherapy remains an active area of investigation, future progress may depend on combining tau-targeted agents with upstream disease-modifying strategies or transitioning to transcript-level interventions better suited to modulate intracellular tau burden.

Nevertheless, the scalability and preventive potential of vaccines continue to drive interest. In contrast to mAb therapy, which is expensive and logistically demanding, vaccines could be deployed in preclinical or at-risk populations, including ApoE4 carriers or individuals with biomarker evidence of early pathology. As such, therapeutic vaccination represents a promising, if still evolving, strategy for AD modification, particularly as an adjunct to other biologics or in preventive contexts.

### 3.3. RNA Therapeutics and Gene-Modifying Approaches: Redefining Targets at the Transcript Level

The past decade has seen a growing shift from protein-directed therapies toward transcript-level interventions in AD. RNA-based therapeutics, including ASOs, siRNAs, and emerging gene therapies, offer the ability to modulate pathogenic gene expression upstream of protein accumulation. These biologics present distinct advantages in accessing intracellular targets and achieving allele- or isoform-specific regulation, especially for diseases with well-defined genetic risk factors.

One of the most advanced RNA therapeutics in AD is BIIB080, an ASO targeting *MAPT* mRNA, designed to reduce production of all tau isoforms [180]. Administered intrathecally, BIIB080 achieved dose-dependent, sustained reductions in CSF total and p-tau in a Phase 1b study, without significant adverse events [181]. This intracellular mechanism may overcome the limitations observed with extracellular tau antibodies. A Phase 2 trial is currently ongoing, and BIIB080 represents a promising proof of concept for transcript-level tau modulation.

Other RNA-based drugs are targeting amyloidogenic pathways. ALN-APP, an investigational siRNA delivered via lipid nanoparticles (LNPs), aims to suppress *APP* expression and thereby reduce Aβ production [182]. It has entered early-phase clinical trials and highlights the growing interest in hepatic or intrathecal RNA delivery systems that circumvent the BBB. Preclinical siRNA and shRNA candidates are also being developed against targets such as *BACE1*, *PSEN1*, and *ApoE4*, often using modified nanoparticles, cell-penetrating peptides, or viral vectors for brain delivery.

Perhaps most notably, the AAV-based gene therapy LX1001 is under clinical investigation for the upregulating of *ApoE2* expression in AD patients [121]. *ApoE4* remains the strongest genetic risk factor for LOAD, and LX1001 introduces an *ApoE2* plasmid to adjust the ratio of *ApoE2* and *ApoE4* via AAV delivery. This therapy has completed a Phase 2 trial, representing a landmark effort to intervene at the genetic level in sporadic AD. Promising results have been reported, including reductions in key biomarkers such as plasma pTau181 and pTau217.

Gene therapy in AD also includes neuroprotective strategies. NT-501, an encapsulated cell implant engineered to secrete ciliary neurotrophic factor (CNTF), is in clinical evaluation for preserving neuronal function [183]. While cell-based delivery bypasses immune barriers and allows long-term release, issues of surgical implantation, host responses, and limited distribution remain challenges.

Despite their promise, RNA and gene therapies face significant hurdles, including delivery efficiency, long-term safety, immune activation, and cost. Nonetheless, these approaches may enable precision treatment for genetically defined subpopulations (e.g., *ApoE4* homozygotes), multi-target regulation, and early-stage intervention before irreversible pathology occurs. As delivery systems improve and companion diagnostics evolve, transcript- and gene-level biologics are poised to reshape the landscape of AD drug development.

### 3.4. Microbiota-Based Biologics and Gut-Brain Axis Interventions

In line with the gut–brain axis hypothesis, biologics targeting microbiota composition and signaling have entered clinical evaluation for AD. Among these, sodium oligomannate (GV-971) represents the most advanced candidate. Approved in China for mild-to-moderate AD, GV-971 is a marine-derived oligosaccharide reported to remodel gut microbiota, reduce peripheral amino acid–derived metabolites that drive neuroinflammation, and consequently alleviate microglial activation [184,185]. While its global phase IV trials are ongoing, GV-971 provides proof-of-concept that microbiota-based modulation can be translated into a therapeutic strategy for AD.

Probiotic formulations are also under clinical investigation. For instance, a study (NCT05145881) tests a microbial consortium in individuals with MCI to evaluate effects on cognition, systemic inflammation, and gut microbiome diversity. Early pilot studies using probiotics such as *Lactobacillus* and *Bifidobacterium* strains have suggested improvements in Mini-Mental State Examination (MMSE) scores and inflammatory markers, although results remain preliminary and heterogeneous [186]. Another experimental avenue is fecal microbiota transplantation (FMT), where case reports and small pilot trials have demonstrated potential cognitive benefits and changes in amyloid-related biomarkers in AD patients, but robust controlled trials are still lacking [187,188].

Despite these advances, challenges remain. Microbiota composition is highly individualized and influenced by diet, geography, and host genetics, complicating the standardization of therapeutic interventions. Moreover, the causal relationship between dysbiosis and AD progression is not fully established, raising questions about whether microbiota modulation addresses cause or consequence. Safety and durability of long-term interventions also require further clarification.

Nevertheless, microbiota-based biologics offer a novel and complementary approach to classical protein- or gene-targeted therapies. By modulating systemic inflammation, metabolic signaling, and BBB function, gut–brain axis interventions could act synergistically with anti-amyloid or anti-tau biologics. As such, microbiota-directed therapies represent a promising frontier in AD drug development, warranting deeper mechanistic investigation and carefully designed clinical trials.

### 3.5. Failure and Lessons Learned from Biologic Trials

The landscape of biologic therapeutics for AD is littered with high-profile failures, many of which have profoundly influenced how the field now designs and interprets clinical trials. Despite elegant mechanisms, favourable preclinical data, and even target engagement in humans, many biologic agents have failed to demonstrate clinical benefit. These outcomes demand not only technical reassessment, but a broader reflection on disease biology and therapeutic timing, as summarized in Table 2.

Among the most notable disappointments was solanezumab, a mAb designed to bind soluble monomeric Aβ, thereby sequestering it before oligomerization and plaque formation. The mechanistic rationale was elegant: prevent the cascade before it begins. In practice, however, trials including EXPEDITION and DIAN-TU showed that although solanezumab increased peripheral Aβ clearance, it had minimal impact on brain amyloid burden, and no measurable effect on cognition or function [189]. This disconnect between peripheral pharmacodynamics and central pathology raised a critical concern—was the target itself insufficient, or had the intervention simply come too late?

Crenezumab, another antibody with higher affinity for oligomeric Aβ, was expected to overcome the limitations of solanezumab. Yet, in both sporadic and genetically determined AD cohorts (including the landmark Colombian *PSEN1* mutation carriers), Crenezumab failed to slow cognitive decline. Post hoc analyses suggested that by the time symptoms manifest, even in familial AD, removing extracellular Aβ may no longer suffice, particularly if tau and neurodegeneration have already gained momentum [190].

The same theme emerged in anti-tau trials. Semorinemab, Gosuranemab, and Zagotenemab all showed some evidence of target engagement, reducing extracellular tau levels in CSF or plasma, but failed to produce clinical improvements in cognition or function. While antibodies can neutralize extracellular tau, the most toxic tau species likely reside intracellularly, where antibodies have limited access. These trials raised an uncomfortable but essential question: Can immunotherapy succeed if the pathogenic process has already shifted from seeding to irreversible neuronal loss?

Equal importance was the realization that biomarker changes do not always translate to clinical benefit. Several agents showed amyloid clearance or tau reduction on PET or CSF assays, yet patients continued to decline [191]. This decoupling implies that molecular pathology may be necessary but not sufficient to drive the clinical syndrome, or that damage becomes self-sustaining beyond a critical threshold.

Across these failures, a few common themes have emerged. First, many trials enrolled patients in mild-to-moderate stages of AD, too late for disease-modifying interventions to reverse established damage. Second, early biologics often lacked precise stratification tools, resulting in heterogeneous trial populations, some of whom may not have had active amyloid or tau pathology. Third, the field’s reliance on traditional cognitive endpoints may have underestimated subtle, early effects better captured by biomarkers.

Crucially, these failures have reshaped how we now approach clinical trials. Biomarker-confirmed inclusion is now standard [192]. Trials increasingly enrol prodromal or preclinical patients. Outcomes integrate imaging, fluid biomarkers, and cognitive composites. And perhaps most importantly, biologics are no longer tested in isolation, they are being paired with more nuanced diagnostics and, increasingly, with complementary therapeutics targeting inflammation, synaptic function, or metabolic resilience.

In retrospect, these failures were not in vain. They clarified the limits of certain targets, exposed the need for earlier intervention, and forced the field to recalibrate its expectations and designs. The modest success of agents like Lecanemab and Donanemab is built on these hard-earned lessons, and points to a more mature, biologically grounded era of drug development in AD.

## 4. Future Perspectives and Challenges

### 4.1. Rethinking Therapeutic Targets Beyond Amyloid and Tau

The long-standing focus on Aβ and tau in AD research has produced both landmark advances and frustrating limitations. Recent approvals of anti-amyloid antibodies such as Lecanemab and Donanemab offer partial validation of the amyloid hypothesis, but their modest efficacy and safety concerns underscore a crucial reality: amyloid and tau alone may not fully account for disease initiation and progression, particularly in sporadic LOAD. As a result, a paradigm shift is underway, toward broader, multidimensional targets that capture the complexity of AD pathophysiology.

Among the most actively explored area is neuroinflammation, which is increasingly recognized not as a secondary response but as a driver of synaptic loss and neuronal dysfunction. Genetic studies have implicated immune-related genes such as *TREM2*, *CD33*, and *INPP5D*, all of which regulate microglial activation states. Therapeutic strategies aimed at modulating microglial phenotype, rather than simply suppressing inflammation, are gaining ground. For example, AL002 (a TREM2 agonist antibody) is currently in Phase 2 trials, aiming to enhance microglial phagocytosis and resilience in early-stage AD [55].

Another emerging area is vascular dysfunction and BBB breakdown. Evidence suggests that BBB leakage may precede and exacerbate amyloid deposition, tau propagation, and neuroinflammation. Agents such as anti-VCAM1 antibodies or small molecules targeting pericyte health aim to restore neurovascular integrity, potentially delaying disease onset or progression [193].

Metabolic and endocrine dysregulation, particularly insulin signalling impairment, also constitutes a promising therapeutic axis. The repurposing of GLP-1 receptor agonists, such as semaglutide [194], originally developed for diabetes, reflects this metabolic angle. These agents are now being evaluated in Phase 3 AD trials, supported by preclinical data demonstrating anti-inflammatory and neuroprotective effects independent of glycaemic control.

Lipid metabolism, especially involving ApoE, presents another compelling target. *ApoE4*, the strongest genetic risk factor for sporadic AD, affects synaptic plasticity, cholesterol transport, and glial function. Therapies aimed at reducing ApoE4 levels (e.g., LX1001) or enhancing ApoE2-like functions are being developed, including RNA-based strategies and receptor modulators. These efforts aim not just to lower risk, but to modify underlying pathobiology in genetically susceptible individuals.

Collectively, these emerging targets reflect a growing appreciation for AD as a multi-system disorder, involving immune, vascular, metabolic, and genetic factors. Future biologic therapies are likely to succeed not through monotherapy, but via strategic combinations that address multiple axes of disease vulnerability. To that end, mechanistically diverse biologics, and the diagnostics needed to guide their use, represent the next frontier in AD drug development.

### 4.2. Overcoming Delivery Barriers to the Brain

One of the most persistent obstacles in AD biologic development is the challenge of delivering large or complex molecules to the brain. Most biologics, such as mAbs and nucleic acids, face significant barriers in crossing the BBB, a highly selective interface that limits the entry of over 98% of potential CNS therapeutics [195]. As a result, many promising biologics fail not because of insufficient potency, but because they cannot reach their intended targets at therapeutic concentrations.

Currently approved anti-amyloid antibodies, such as Lecanemab and Donanemab, require regular intravenous administration, with systemic distribution and only a small fraction penetrating the CNS. This results in high treatment burdens, risk of peripheral side effects, and increased healthcare costs. Moreover, the need for intensive MRI monitoring, primarily to detect ARIA, underscores the delicate balance between efficacy and safety in CNS-targeted biologics.

To overcome these limitations, several innovative delivery strategies are under development. Among the most promising are LNP platforms, which have gained clinical validation in mRNA vaccine delivery. When tailored with cell-penetrating peptides (CPPs) or surface ligands that recognize brain endothelial receptors (e.g., transferrin or insulin receptor), LNPs may facilitate receptor-mediated transcytosis across the BBB. Furthermore, intranasal administration of nanoparticle-encapsulated therapeutics bypasses systemic circulation altogether, offering a non-invasive route to the brain via the olfactory and trigeminal pathways [143,152].

AAV vectors, widely used in gene therapy, offer another route for durable CNS delivery. While conventional systemic AAV administration often leads to hepatic sequestration and immune clearance, recent innovations in capsid engineering and direct intracerebroventricular (ICV) or intrathecal injection (IV) have enhanced CNS tropism. However, concerns around immunogenicity, manufacturing cost, and limited dosing capacity remain.

Other emerging approaches include focused ultrasound (FUS) with microbubbles to transiently disrupt the BBB [196], exosome-based delivery systems, and biodegradable polymeric nanoparticles (PNPs). Each has shown promise in animal models, but translation to clinical-scale human application remains in early stages.

In essence, solving the delivery challenge is not merely a technical hurdle, it is a prerequisite for translating biological insight into therapeutic success. As more biologics target intracellular or gene-level mechanisms, delivery platforms will need to balance efficiency, safety, and patient acceptability. Ultimately, innovations in delivery may prove just as critical as target selection in shaping the future of AD therapy.

### 4.3. Designing Trials for Prevention and Precision

The limited efficacy of many AD therapeutics, especially biologics, has increasingly been attributed not to the failure of mechanisms per se, but to late timing of intervention. By the time clinical symptoms emerge, neurodegeneration is often well underway, with widespread synaptic loss, glial dysfunction, and tau pathology. This has shifted the therapeutic paradigm toward early-stage or even pre-symptomatic intervention, ushering in a new era of prevention-oriented and precision-guided trial design.

A cornerstone of this shift is the use of biomarkers to detect preclinical disease [197]. Advanced imaging, like amyloid-PET and tau-PET; CSF biomarkers, including Aβ42/40 ratio and p-tau; and increasingly plasma biomarkers, such as p-tau181, p-tau217, and neurofilament light chain (NfL), now enable the identification of individuals with pathological changes years before cognitive symptoms appear. These markers not only facilitate early diagnosis, but also allow for pathology-confirmed inclusion criteria, as exemplified by the CLARITY-AD and TRAILBLAZER-ALZ studies [198].

Genetic risk factors, particularly ApoE4 carrier status, are also being used to enrich trial populations most likely to benefit from specific interventions, or to stratify for adverse event risks such as ARIA. Trials like the AHEAD 3–45 and API Generation Study have embraced this genotype-based approach, enrolling cognitively normal individuals at elevated risk for AD. Meanwhile, gene-targeted biologics such as LX1001 specifically address genetically defined subgroups, highlighting the potential for genotype-guided therapy.

Innovations in trial design are equally important. Adaptive designs allow for real-time modification of trial parameters, such as dosage and patient subgroup allocation, based on interim data. Platform trials test multiple drugs in parallel within a shared infrastructure, improving efficiency and comparability. These flexible models may be particularly valuable for AD, where biological heterogeneity and slow progression demand long, costly studies.

Outcome measures are also evolving. In early-stage populations, conventional cognitive scales often lack sensitivity to subtle change. Composite endpoints that integrate biomarker shifts, digital cognitive assessments, and functional measures are being developed to more accurately capture early therapeutic effects. Moreover, fluid biomarkers are increasingly used as surrogate endpoints, potentially shortening trial durations and enabling earlier regulatory evaluation [12,199,200].

Together, these innovations signal a shift from the one-size-fits-all approach of earlier decades toward a personalized, stage-sensitive therapeutic model. By selecting the right patients, at the right time, and measuring the right outcomes, prevention- and precision-oriented trials hold the promise of transforming AD drug development, especially for biologics that may act most effectively before irreversible damage occurs.

### 4.4. Ethical, Regulatory, and Access Considerations

As biologic therapies for AD move toward broader clinical use, ethical and practical challenges become increasingly prominent. High treatment costs, complex administration protocols, and biomarker-based eligibility raise concerns about equity and access. Intravenous antibodies like Lecanemab require frequent infusions and MRI monitoring, limiting feasibility in many health systems. Gene and RNA therapies, such as LX1001 or BIIB080, pose additional hurdles in terms of regulatory oversight, long-term safety, and manufacturing complexity.

Efforts to intervene in the preclinical stage also introduce dilemmas. Early biomarker or genetic risk disclosure, particularly in asymptomatic individuals, can trigger psychological distress, discrimination risks, and challenges in informed consent [201]. Identifying who should be screened, and when, will require careful ethical and public health frameworks.

Moreover, as diagnostics and therapies become increasingly commercialized, regulators face the task of balancing innovation with transparency and affordability. Surrogate endpoints, like amyloid clearance, are gaining regulatory acceptance, but their relationship with long-term clinical outcomes remains under scrutiny.

In the long run, biologic progress must be matched by policy mechanisms that promote fair access, ensure safety monitoring, and support sustainable implementation. Without such frameworks, scientific advances may risk deepening, rather than alleviating, disparities in dementia care.

## Figures and Tables

**Table 1:** Ongoing Clinical Trials of Biologic Therapeutics for Alzheimer’s Disease: Targets, Mechanisms, and Development Stages.

Drug	Target	Biologic Type	Mechanism of Action	Dosage	Clinical Trial Phase	AD Stage	Sponsor
Aducanumab	Aβ aggregates	mAb	FcγR-mediated microglial clearance of aggregated Aβ	IV: 10 mg/kg	Approved/Phase IV (NCT04241068, NCT02484547)	Early/mild AD	Biogen, Cambridge, MA, USA
CM383			Binds aggregated Aβ to promote clearance and reduce plaque-associated neurotoxicity	/	Phase I (NCT06619613)	MCI/mild AD	Keymed Biosciences, Chengdu, China
IBC-Ab002			Targets aggregated Aβ to enhance clearance through Fc-mediated phagocytosis	/	Phase I (NCT05551741)	Early AD	ImmunoBrain Checkpoint, Rehovot, Israe
Lecanemab	Aβ protofibrils		Binds protofibrillar Aβ to reduce plaque formation and neurotoxicity	IV: 10 mg/kg	Approved/Phase IV (NCT03887455, NCT01767311)	Early AD	Eisai, Tokyo, Japan
Sabrinetug			Binds Aβ protofibrils to neutralize toxicity and facilitate immune clearance	IV: 35 or 50 mg/kg	Phase I & II (NCT06335173)	Early AD	Acumen Pharmaceuticals, Greater Boston, MA, USA
ALIA-1758			Binds Aβ protofibrils to promote clearance and inhibit neurotoxicity	/	Phase I (NCT06406348)	Early AD	AbbVie, North Chicago, IL, USA
APNmAb005			Binds to amyloid protofibrils to neutralize neurotoxic aggregates	IV: from 5 to 70 mg/kg	Phase I (NCT05344989)	Early AD	APRINOIA Therapeutics, Suzhou, Chi
MK-2214			Binds protofibrillar Aβ to facilitate immune clearance	/	Phase I (NCT05466422)	Early AD	Merck & Co. (MSD), Rahway, NJ, USA
Donanemab	Pyroglutamate-modified Aβ		Targets modified plaques to clear established amyloid	IV: 1400 mg/4 weeks	Approved/Phase IV (NCT04437511, NCT05026866)	Early/mild AD	Eli Lilly, Indianapolis, IN, USA
Remterneutug			Targets pyroglutamate-modified Aβ to remove established amyloid plaques	/	Phase III (NCT06653153)	Early symptomatic AD	Eli Lilly, Indianapolis, IN, USA
ABBV-916	N3 pyroglutamate Aβ		Binds N-terminal truncated Aβ (pyroglutamate) to clear plaques	/	Phase II (NCT05291234)	Early AD	AbbVie, North Chicago, IL, USA
LY3954068	Aβ oligomers		Binds toxic Aβ oligomers to neutralize neurotoxicity	/	Phase I (NCT06297590)	Early AD	Eli Lilly, Indianapolis, IN, USA
Bepranemab	Extracellular full-length tau		Targets extracellular tau to prevent propagation of pathological species	/	Phase II (NCT04867616)	Early AD	UCB Biopharma, Brussels, Belgium
E2814	Tau MTBR (microtubule-binding region)		Binds extracellular MTBR-tau to inhibit seeding and propagation of pathogenic tau species, clearance mediated by microglia	IV: 750 or 1500 mg	Phase II (NCT04971733, NCT06602258 with Lecanemab)	Early/sporadic AD & DIAD	Eisai, Toky, Japan
BMS-986446			Binds MTBR-tau to inhibit tau aggregation and spread	/	Phase II (NCT06268886)	Early AD	Bristol-Myers Squibb, New York, NY, USA
PMN310	Misfolded tau aggregates		Targets pathological tau conformers to block downstream neurodegeneration	IV: from 175 to 2800 mg	Phase I (NCT06105528)	MCI/Early AD	ProMIS Neurosciences, Toronto, ON, Canada
JNJ-63733657	p-tau		Binds p-tau to inhibit extracellular spread and promote clearance	/	Phase II (NCT04619420)	Early AD	Janssen, Raritan, NJ, USA
Trontinemab	Tau oligomers		Targets extracellular tau oligomers to prevent propagation	/	Phase I (NCT04639050)	Early/mild AD	Roche, Basel, Switzerland
SHR-1707	TREM2		Activates microglial TREM2 signaling to promote phagocytosis and reduce inflammation	/	Phase I & II (NCT06199037)	Early AD	Hengrui Pharma, Shanghai, China
AL101	Sortilin receptor (elevates progranulin, PGRN)		Downregulates Sortilin to increase PGRN levels, enhancing lysosomal function and neuroprotection	/	Phase II (NCT06079190)	Early AD	GSK, London, UK
Foralumab	CD3 on T lymphocytes		Modulates neuroinflammation via nasal anti-CD3 immunotherapy	/	Phase II (NCT06489548)	Early AD	Brigham and Women’s Hospital, Boston, MA, USA
BIIB080	*MAPT* mRNA (tau)	ASO	Reduces tau production by degrading *MAPT* mRNA via RNase H–dependent ASO mechanism, targets intracellular tau broadly	/	Phase II (NCT05399888)	MCI/mild AD dementia	Biogen, Cambridge, MA, USA
ION269			Reduces tau expression by degrading *MAPT* mRNA via RNase H mechanism	/	Phase I (NCT06673069)	Mild AD	Ionis, Carlsbad, CA, USA
NIO752			Reduces tau protein production via intrathecal ASO targeting *MAPT* mRNA	/	Phase I (NCT05469360, NCT06372821)	Early AD/MCI	Novartis, Basel, Switzerland
ALN-APP	*APP* mRNA	siRNA (LNP)	Suppresses *APP* expression to reduce Aβ production	/	Phase I & II (NCT05231785, NCT06393712)	Early AD (presymptomatic/MCI)	Alnylam, Cambridge, MA, USA
LY3954068	*MAPT* mRNA (tau)	siRNA	Reduces tau production by degrading *MAPT* mRNA	/	Phase I (NCT06297590)	Early AD	Eli Lilly, Indianapolis, IN, USA
LX1001	*ApoE4* homozygotes	*ApoE2* mRNA	Increase expression of *ApoE2*	/	Phase II (NCT03634007)	Mild to moderate AD	Lexeo Therapeutics, New York, NY, USA
Leuprolide	GnRH receptor (GNRHR)	Hormonal analog	Modulates sex hormones to potentially slow neurodegeneration in postmenopausal women	/	Phase II (NCT03649724)	MCI/mild AD	Weill Cornell, New York, NY, USA
Insulin	CNS insulin receptor	Peptide hormone	Improves brain glucose metabolism and cognition via central insulin signalling	/	Phase II/III (NCT01767909)	MCI/mild AD	USC, Los Angeles, CA, USA
Semaglutide	GLP-1 receptor	Peptide agonist	Modulates insulin signalling, reduces neuroinflammation, promotes neuroprotection	Oral: 14 mg/day	Phase III (NCT04777396, NCT04777409)	Early AD	Novo Nordisk, Bagsværd, Denmark
Posiphen	Aβ antigen (adjuvant only)	TLR9 agonist adjuvant	Enhances immune response to co-administered Aβ vaccine antigens via TLR9 activation	Oral: 80 mg/day	Phase I (NCT04524351)	Mild AD	Annovis Bio, Berwyn, PA, USA
AV-1959D	Aβ1–11 epitope	DNA Vaccine	Induces polyclonal antibodies against Aβ to clear plaques	/	Phase I (NCT05642429)	Early AD	IMM, Oxford, UK
ACI-24.060	Aβ oligomers & pyroglutamate Aβ	Active immunization (vaccine)	Induces polyclonal antibodies targeting pathogenic Aβ species, promoting immune-mediated clearance	/	Phase II (NCT05462106)	Prodromal AD & Down syndrome	AC Immune SA, Lausanne, Switzerland
ALZ-101	Aβ1–42 peptide		Elicits antibodies against Aβ1–42 to promote clearance	/	Phase I/II (NCT05328115)	Early AD	Alzinova AB, Mölndal Sweden
JNJ-2056	p-tau		Elicits antibodies against phosphorylated tau epitopes to inhibit intracellular aggregation	/	Phase I/IIa (NCT04445831)	MCI/mild AD	AC Immune SA, Lausanne, Switzerland
BCG Vaccine	Trained innate immunity (macrophages)	Live attenuated vaccine	Boosts innate immune surveillance to mitigate neuroinflammation	/	Phase I/II (NCT05004688)	Early AD	Massachusetts General Hospital, Boston, MA, USA
Aldesleukin	IL-2 receptor on Tregs	Recombinant cytokine	Low-dose IL-2 selectively expands regulatory T cells to reduce neuroinflammation	/	Phase IIa (NCT05468073)	Early AD	Centre Hospitalier St Anne, Paris, France
Sargramostim	Granulocyte-macrophage colony-stimulating factor (GM-CSF) receptor		Stimulates innate immune cells to enhance Aβ clearance and neuroprotection	/	Phase II (NCT04902703)	Mild-to-moderate AD	University of Colorado, Denver, CO, USA
ExPlas	Plasma factors	Plasma exchange biologic	Transfuses young plasma components to support neuronal health	IV: 200 mL/week	Phase IIa (NCT05068830)	Mild AD to moderate	Norwegian University of Science and Technology, Trondheim, Norway
Recombinant Human Serum Albumin	Circulating Aβ	Plasma protein infusion	Alters peripheral Aβ dynamics to promote central Aβ clearance	/	Phase I (NCT06489015)	Mild to moderate AD	Protgen, Beijing, Chin
XPro1595	Soluble TNFα	Dominant-negative TNF biologic	Inhibits soluble TNFα to reduce neuroinflammation and synaptic loss	SC: from 0.3 to 1.0 mg/kg	Phase II (NCT03943264)	Mild AD	INmune Bio, Boca Raton, FL, USA
ADEL-Y01	TGF-β1 pathway	Stem cell-derived biologic	Adipose-derived stem cell secretome modulates neuroinflammation and synaptic repair	IV: 20 mg/kg	Phase Ia/Ib (NCT06247345)	Mild to moderate AD	ADEL, Seoul, South Korea
Lomecel-B	MSC-secreted immunomodulatory and neurovascular factors	Allogeneic MSC therapy	Anti-inflammatory, pro-vascular, tissue repair, immunomodulatory, and low immunogenicity	/	Phase IIa (NCT05233774)	Mild AD	Longeveron, Miami, FL, USA
GV-971	Gut microbiota	Sodium oligomannate	Reconditions the dysbiosis of gut microbiota, inhibits the inflammatory response in the brain	Oral: 900 mg/day	Phase IV (NCT05058040)	Mild to moderate AD	Green Valley, Shanghai, China
Probiotics	Gut microbiota	Microbial consortia/dietary	Modulates gut–brain axis to reduce systemic inflammation and improve cognitive function	/	Phase II (NCT05145881)	MCI	Hsieh-Hsun Ho, Taiwan, China

Clinical trial phase and NCT identifiers were obtained from *ClinicalTrials.gov*.

**Table 2:** Discontinued or Failed Biologic Therapeutics in Alzheimer’s Disease: Clinical Trial Phases, Targets, and Reasons for Termination.

Drug	Target	Biologic Type	Mechanism of Actin	Dosage	Clinical Trial Phase (At Failure)	Reason for Failure/Outcome	Sponsor
Gantenerumab	Fibrillar Aβ	mAb	Clears fibrillar Aβ via microglial activation	/	Phase III (GRADUATE 1: NCT03444870, GRADUATE 2: NCT03443973)	Despite significant plaque reduction, no cognitive benefit was observed in interim analyses, leading to early termination.	Roche, Basel, Switzerland
Bapineuzumab			Targets Aβ plaques via Fc-mediated phagocytosis	IV: 0.5 or 1.0 mg/kg	Phase III (Studies 301/302: NCT00667810, NCT00667824)	Failed to meet co-primary cognitive and function endpoints in ApoE4 carriers & non-carriers, incidence of ARIA, all trials subsequently discontinued	Pfizer, New York, NY, USA
Solanezumab	Soluble Aβ		Enhances peripheral clearance of soluble Aβ	IV: from 400 to 1600 mg/4 weeks	Phase III (EXPEDITION 1–3: NCT02008357, A4: NCT02008357)	Failed to meet primary cognitive endpoints in mild-to-moderate and preclinical AD, subgroup analyses showed only marginal trends without statistical power.	Eli Lilly, Indianapolis, IN, USA
Ponezumab			Peripheral sink effect, targets Aβ to enhance systemic clearance	IV: 7.5 or 10 mg/kg	Phase II (NCT00945672)	No cognitive or biomarker benefit in mild-to-moderate AD patients	Prizer, New York, NY, USA
Crenezumab	Aβ oligomers		Binds oligomeric Aβ, reduced Fc effector activity (IgG4)	IV: 60 mg/kg every 4 weeks	Phase III (NCT03491150)	Terminated after futility analyses revealed no significant clinical benefit in prodromal/mild AD, also failed in the Alzheimer’s Prevention Initiative (API) for autosomal dominant AD.	Roche, Basel, Switzerland
Gantenerumab	Aggregated Aβ		Anti-Aβ monoclonal antibody	SC: from 105 to 1200 mg/4 weeks	Phase 3 (NCT01224106)	Trial completed with no follow-up, development not continued	Roche, Basel, Switzerland
GSK933776			Binds plaque-forming Aβ to lower CNS Aβ without ARIA	IV: from 0.001 to 10 mg/kg	Phase I (NCT00459550, NCT01424436)	Engaged target and altered Aβ levels, but no subsequent efficacy trials, likely discontinued	GSK, London, UK
Gosuranemab	Extracellular N-terminal tau		Binds N-terminal tau to block cell-to-cell propagation	/	Phase II (TANGO: NCT03352557)	Trial failed to demonstrate clinical benefit despite target engagement, program discontinued.	Biogen, Cambridge, MA, USA
Semorinemab			Targets N-terminal tau to inhibit spreading of pathology	/	Phase II (Tauriel: NCT03289143, Lauriet: NCT03828747)	No significant slowing of cognitive or functional decline observed in mild-to-moderate AD patients.	Genentech, South San Francisco, CA, USA
AL002	TREM2		Activates microglia via TREM2 to enhance Aβ clearance and modulate neuroinflammation	/	Phase II (NCT05744401)	Parent study failed to meet the primary endpoint and there were no treatment effects that favored AL002 on secondary clinical and functional endpoints.	Alector, South San Francisco, CA, USA
AN-1792	Full-length Aβ_1–42_	Peptide vaccine	Induces polyclonal anti-Aβ antibody response	/	Phase II (NCT00021723)	Induced meningoencephalitis in ~5% of participants due to autoimmune T-cell response, program halted for safety reasons.	Janssen, South San Francisco, CA, USA
ACC-001	Aβ1–7 peptide (with QS-21 adjuvant)		Vaccine targeting Aβ1–7 with QS-21 adjuvant	/	Phase II (NCT01284387)	Terminated due to injection-site reactions and lack of efficacy	Janssen, South San Francisco, CA, USA
ABvac40	C-terminal Aβ_40_ epitope		Induces antibodies targeting Aβ40 to prevent aggregation	/	Phase II (NCT03113812)	Phase II results unpublished, program status unclear, likely discontinued	Araclon Biotech, Zaragoza, Spain
UB-311	Aβ1–14 (soluble and aggregated forms)		Induces Th2-skewed anti-Aβ antibody response	/	Phase IIa (NCT03531710)	Trial completed, no publicly reported efficacy outcomes since 2021	United Neuroscience Ltd. Cork, Ireland
AADvac1	Truncated pathological tau		Induces antibodies against misfolded tau protein	/	Phase II (NCT02579252)	No significant slowing of cognitive decline, biomarker changes modest, development halted or stalled	AXON Neuroscience SE, Larnaca, Cyprus
BIIB092	N-terminal extracellular tau		Binds extracellular tau to block seeding and spread	/	Phase II (NCT03352557)	Failed to slow clinical decline despite tau binding, development discontinued	Biogen, Cambridge, MA, USA
RO7105705			Blocks tau propagation in extracellular space	/	Phase II (NCT03828747)	Modest biomarker changes but failed to slow clinical progression	Genentech, South San Francisco, CA, USA
Lu AF20513	Multi-epitope Aβ	DNA vaccine	Targets multiple Aβ epitopes	/	Phase I (NCT03668405)	Study completed with no publication or further clinical development	H. Lundbeck A/S, Copenhagen, Denmark
CAD106.	Aβ1–6	Active vaccine	Induces Aβ antibodies while avoiding T-cell response	IM: 450 µg	Phase II/III (NCT02565511)	Did not meet cognitive endpoints in patients with prodromal AD	Novartis, Basel, Switzerland
Octagam IVIG	Polyvalent antibodies	IV immunoglobulin therapy	Hypothesized to clear Aβ/Opsonize for immune-mediated clearance	/	Phase III (NCT01561053)	Failed to meet cognitive or functional endpoints in mild-to-moderate AD	Instituto Grifols, Barcelona, Spain

Clinical trial phase and NCT identifiers were obtained from *ClinicalTrials.gov*.

## Data Availability

No new data were created or analyzed in this study. Data sharing is not applicable to this article.
